# ALTERNATIVE TECHNIQUE FOR CHOLECYSTECTOMY COMPARABLE TO SINGLE PORT

**DOI:** 10.1590/0102-6720201700010015

**Published:** 2017

**Authors:** Carlos SABBAG, Ana BLITZCKOW

**Affiliations:** Hospital Santa Cruz, Hospital Vita Batel and Clínica Los Angeles (Santa Cruz Hospital, Vita Batel Hospital and Los Angeles Clinic), Curitiba, PR, Brazil

**Keywords:** Cholecystectomy, laparoscopic. Cholecystitis. Cholelithiasis. Minimally invasive surgical procedures

## Abstract

**Background:**

With the advancement of laparoscopic surgery, new techniques have been proposed and disseminated in order to reduce the surgical aggression and get better cosmetic results.

**Aim:**

To present alternative technique for videocholecystectomy comparable to single port technique using conventional material for laparoscopic surgery.

**Method:**

Introduction of laparoscopic devices using two incisions; gallbladder traction with thread, exposition of Calot triangle, and ligature of cystic pedicle with polymer clips.

**Results:**

Nine operations were carried out with this method, without complications and no increase in operative time, being compared to conventional videocholecistectomy, however vastly superior in aesthetic results.

**Conclusion:**

The technique is feasible, reproducible, showing benefits to patient´s safety

## RESUMO

**Racional:**

Com o avanço da cirurgia laparoscópica, novas técnicas têm sido propostas e difundidas com o objetivo de diminuir a agressão cirúrgica e obter melhores resultados estéticos.

**Objetivo:**

Apresentar técnica alternativa para videocolecistectomia similar à técnica de single port, contudo utilizando material convencional para cirurgia laparoscópica.

**Método:**

Procedimento de videocolecistectomia com uso de duas incisões, exposição do trígono de Calot por tração da vesícula biliar com fio e ligadura dos elementos do hilo cístico com clipes de polímero.

**Resultado:**

Foram realizadas nove operações com esse método, não se observando complicações e nem aumento do tempo operatório em relação à videocolecistectomia convencional, contudo com resultado estético grandemente superior.

**Conclusão:**

A técnica é factível, reprodutível e mostra benefícios e segurança ao paciente.

## INTRODUCTION

The interest in minimizing surgical invasiveness has increased, prompted by the evolution of laparoscopic surgery and the development of new technologies. Aesthetic results and the time to return to work have been optimized. The literature amply demonstrates the benefits of minimally invasive surgery. Surgeons have developed alternative methods to perform increasingly less invasive procedures, while minimizing the number of ports. Some of these techniques have been reported and are currently under evaluation, or in the development process. Natural orifice transluminal endoscopic surgery (NOTES) and single port technique performed through a transumbilical incision are examples of the search for the ideal laparoscopic operation. These new procedures reduce both the number of incisions and surgical invasiveness; however, they are associated with increased cost and the risks of complications; moreover, new techniques must be learned. 

The aim of this study was to present an alternative technique using conventional videolaparoscopy instruments to perform a procedure comparable to single-port surgery. 

## METHOD

### Technique

**FIGURE 1:**
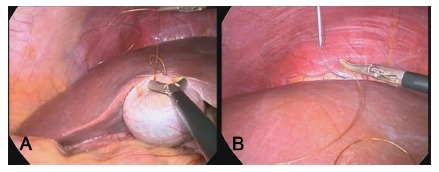
A) Fixation of the fundal sac using 2-0 chromic catgut suture with a gastrointestinal needle; B) catgut suture is introduced into the pink-hubbed needle (1.2 mm diameter) positioned in the intercostal space

The procedure begins conventionally, with a 15-mm transumbilical incision, insertion of a Veress needle, pneumoperitoneum, and introduction of a 10-mm trocar. A second 5-mm trocar is introduced through the same incision to the right of the first, under direct optical observation, while a third trocar with the same diameter is placed on the left side, at the epigastric level. A 2-0 chromic catgut suture (gastrointestinal needle) is introduced through the 10-mm trocar (using a reducer) with needle correction and a loop at the end; this suture will be attached to the fundus of the gallbladder, and appropriate traction is achieved by passing the needle through the loop to close it (Figure 1A). The thread is trimmed to a length 2-3 cm from the needle, which is removed through the 5-mm trocar. This is followed by puncture of the intercostal space with a pink-hubbed needle (40x12), from the right midclavicular to the anterior axillary lines, between the 7^th^ and 9^th^ ribs (according to the surgeon's preference), being careful to avoid the neurovascular bundle. The catgut suture is then threaded into the pink-hubbed needle (1.2 mm diameter, Figure 1B) positioned in the intercostal space, externalized, and pulled, fully exposing the gallbladder and Calot's triangle. This method allows greater mobility, depending on the location of chest wall puncture.

**FIGURE 2:**
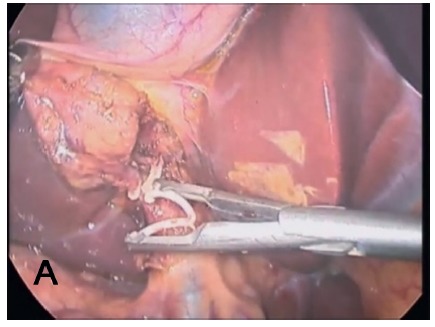
Ligature of the duct and cystic artery using polymer clips (green cartridge/medium)

The dissection of the cystic pedicle is performed in the usual manner, while duct and cystic artery ligature is performed with polymer clips (green cartridge/medium); the clipper is inserted through the 5-mm port (Figure 2).

Conventional methods are used in gallbladder dissection from the fossa, hemostasis, and revision. The extraction of the bladder from the abdomen is done by briefly backing out the optics, followed by use of an auxiliary clamp to direct the sac into the 10-mm trocar, always under direct observation. The set is externalized en bloc through the umbilicus. The 5-mm trocar positioned to the left of the epigastrium enables angled access, as well as prior evacuation of biliary fluid by puncture and aspiration. After extraction of the gallbladder through the umbilicus, the 10-mm trocar is repositioned with optics for final revision. 

## RESULTS

**FIGURE 3:**
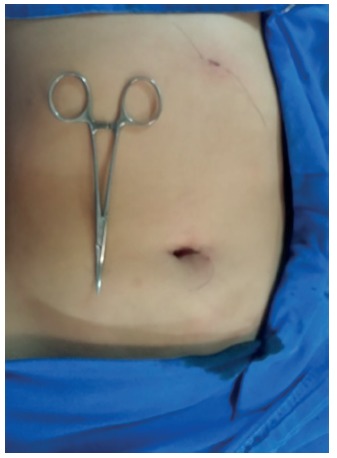
Aesthetic result, superior to conventional videocholecystectomy

Nine operations were performed using this method, without additional complications or an increase in operative time; the aesthetic result and minimally invasive approach to the abdominal wall proved vastly superior to conventional videocholecystectomy (Figure 3).

## DISCUSSION

The technique proposed herein demonstrates that basic laparoscopic instruments can be used. The end result is comparable to that of a single-port procedure; the difference lies in the placement of an extra 5-mm port in the epigastrium - which is often necessary in a single-port operation. Gallbladder traction via intercostal puncture is shown to be safe, with similar or better results than with the traditional use of forceps in the right flank. This method is thought to be safer than using nylon thread for fixation to the abdominal wall, as it reduces trauma and risk of complications.

**FIGURE 4:**
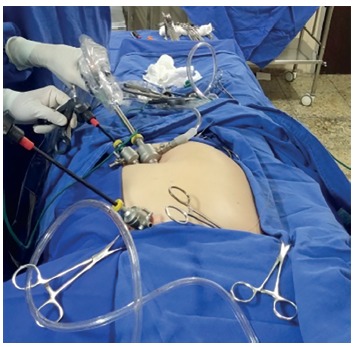
Setup with two trocars in the umbilicus, allowing the surgeon's left hand to hold the gallbladder wall

There is no description of this procedure in the literature. Accordingly, after researching various materials, it was decided that 2-0 chromic catgut was the optimal choice of suture (gastrointestinal needle), along with a pink-hubbed needle (40x12). The chromic thread coating allows passage through the needle bore without difficulty. Moreover, this technique can be used in other procedures such as gynecological operations, preparation for intra-abdominal mesh stapling in incisional hernia repair, and intestinal anastomoses. The use of a 5-mm trocar requires polymer clips, since use of traditional clips larger than this trocar size can result in a less secure ligature (polymer clips contain locking devices). The authors have used thread ligatures before (intra- and extracorporeal knots); however, there is a considerable increase in the operative time, and the ligature appears to be less effective compared to this type of clip. The added cost is not significant. The placement of two trocars in the umbilicus does not create additional difficulty, and allows the surgeon's left hand to hold the bladder wall; specific training is not required, only adaptation (Figure 4). It is thought that this method presents the following advantages over a single-port operation: 1) exposure of Calot's triangle comparable to that in conventional laparoscopic cholecystectomy (which makes the procedure safe); 2) very satisfactory aesthetic outcome; 3) reduced trauma to a small umbilicus, comparable to that in conventional laparoscopic cholecystectomy; and 4) cost comparable to that of a traditional operation; moreover, only standard instruments are used.

## CONCLUSION

The technique presented is feasible, reproducible, and has advantages; it is safe for patients, does not significantly increase costs, uses only conventional laparoscopic instruments, and provides excellent aesthetic results.
